# Structural characterization of CA1462, the *Candida albicans *thiamine pyrophosphokinase

**DOI:** 10.1186/1472-6807-8-33

**Published:** 2008-07-24

**Authors:** Sébastien Santini, Vincent Monchois, Nicolas Mouz, Cécile Sigoillot, Tristan Rousselle, Jean-Michel Claverie, Chantal Abergel

**Affiliations:** 1 Information Genomique et Structurale, UPR2589, Parc Scientifique de Luminy, 13288, Marseille cedex 09, France; 2 Protein'eXpert, 7 Parvis Louis Néel, BP50 38040 Grenoble, France

## Abstract

**Background:**

In search of new antifungal targets of potential interest for pharmaceutical companies, we initiated a comparative genomics study to identify the most promising protein-coding genes in fungal genomes. One criterion was the protein sequence conservation between reference pathogenic genomes. A second criterion was that the corresponding gene in *Saccharomyces cerevisiae *should be essential. Since thiamine pyrophosphate is an essential product involved in a variety of metabolic pathways, proteins responsible for its production satisfied these two criteria.

**Results:**

We report the enzymatic characterization and the crystallographic structure of the *Candida albicans *Thiamine pyrophosphokinase. The protein was co-crystallized with thiamine or thiamine-PNP.

**Conclusion:**

The presence of an inorganic phosphate in the crystallographic structure opposite the known AMP binding site relative to the thiamine moiety suggests that a second AMP molecule could be accommodated in the *C. albicans *structure. Together with the crystallographic structures of the enzyme/substrate complexes this suggests the existence of a secondary, less specific, nucleotide binding site in the *Candida albicans *thiamine pyrophosphokinase which could transiently serve during the release or the binding of ATP. The structures also highlight a conserved Glutamine residue (Q138) which could interact with the ATP α-phosphate and act as gatekeeper. Finally, the TPK/Thiamine-PNP complex is consistent with a one step mechanism of pyrophosphorylation.

## Background

Our laboratory runs a structural genomics project (PROFUN [1]) targeting fungal protein-coding genes in search of new anti-fungal targets. The main goal of this project is to quantitatively express the selected *Saccharomyces cerevisiae *and *Candida albicans *genes products to characterize them functionally and structurally as well as explore their potential as new drug targets. The genes selected in *Candida albicans *are of two types: 1) they have orthologous genes in *Saccharomyces cerevisiae *that are essential or 2) they are conserved in pathogenic fungal genomes and can be absent from the non-pathogenic yeast *Saccharomyces cerevisiae*.

*Candida albicans *is a diploid organism with eight sets of homologous chromosomes and a genome size of about 32 Mb. This pathogen is part of the normal microflora of the human gastrointestinal tract or oropharynx and is responsible for various nosocomial infections, potentially lethal in immunocompromised patients or patients in the intensive care unit. The CA1462 protein is encoded by a gene conserved across all known fungal genomes. It shares 36% sequence identity with *Saccharomyces cerevisiae *YOR143C [2], a Thiamine pyrophosphokinase (TPK, EC 2.7.6.2) essential for fungus growth and survival [3]. This enzyme is also essential in *S. pombe *[4]. The production of thiamine pyrophosphate (TPP), catalyzed by TPK, is critical for both catabolic and anabolic cellular processes. In vertebrates, a deficiency in thiamine synthesis is linked to neurological diseases such as beriberi and Wernicke encephalopathy [5]. In all synthesizing organisms, thiamine (vitamin B1) is produced from phosphorylated HET (4-methyl-5-(β-hydroxymethyl)thiazole) and HMP (2-methyl-4-amino-5-hydroxymethyl-pyrimidine) that are condensed into thiamine phosphate. In yeast, this thiamine phosphate is then hydrolyzed into thiamine before pyrophosphorylation by the TPK that catalyzes the transfer of a pyrophosphate (PPi) from ATP to thiamine, resulting in AMP and TPP [6].

This article reports the enzymatic characterization as well as the structure determination of the *Candida albicans *CA1462 Thiamine pyrophosphokinase in complex with both thiamine and thiamine-PNP (TPNP). We also present a comparison of CA1462 with the structure of the mouse TPK ternary complex with pyrithiamine pyrophosphate and AMP (PDB id 2F17) [7], with the TPK *S. cerevisiae *structure (PDB id 1IG0) [8] and with sequences from other species.

## Methods

### General cloning strategy

The cDNAs corresponding to the TPK genes were amplified by PCR from their respective genomic DNAs (CA1462: *Candida albicans *strain NIH 3147 ATCC number MYA-2876D; YOR143C:*Saccharomyces cerevisiae *strain NRRL Y-53 ATCC number 2601D-5). Gene cloning was performed using the ligation-independent cloning (LIC) method based on ligation of sticky ends generated by T4 DNA polymerase [9]. For the purpose of the PROFUN project, we constructed a specific expression vector, pSF-04 (see Additional file 1) Patent [10], compatible with LIC cloning using the following procedure. The *Eco*RI+*Bam*HI region of PQE60 was inserted in pQE80L vector where *Xho*I, *Nco*I and *Mfe*I sites have been mutated. Then the LacZ encoding gene was PCR amplified using primers containing flanking sequences (represented in bold) used for LIC cloning:

5'-*CCATGG*CTcatcaccatcaccatcacGGG**CATCACCATCAATTG**, forward primer containing the coding sequence (lower case) for a poly-histidine tag,

5'-*GGATCCCTCGAG*TTAG**TCACCATCCAATTG**, reverse primer.

In the absence of an insert, the pSF-04 expression vector expresses the *lacz *gene, while the in frame insertion of the resulting PCR products into *Nco*I+*Bam*HI sites deactivates the gene thus allowing the easy detection of the parental vector [10]. In addition; the GFP encoding gene was PCR-amplified using primers containing flanking sequences 5'-*CTCGAG***GCGGCCG **and 5'*-GGATCC*ATTAT**GCGGCCGC**. This PCR product was inserted at the 3'end of the LacZ encoding gene into *Xho*I+*Bam*HI sites yielding the pSF-04 expression vector that expresses the GFP in C-terminal fusion with the targeted gene. *Not*1 restriction sites (GCGGCCGC) flanking the GFP encoding gene allows its removal [10].

### Cloning and expression screening of CA1462 and YOR143C TPK genes

PCR amplifications were performed using primers specific for CA1462 and YOR143C TPK genes preceded by 5'-CATCACCATCAATTG (Direct primer) and 5'-TCACCATCCAATTG (Reverse Primer) together with purified *Candida albicans *and *Saccharomyces cerevisiae *genomic DNA as template. The PCR products were directly purified using the NucleoSpin Extract kit (Macherey Nagel). Then 0.2 pmol of the purified PCR product was treated with T4 DNA polymerase in the presence of 2.5 mM of dCTP for 30 min. at 22°C before inactivating the enzyme by heating 20 min at 75°C. In a parallel procedure, the expression vector, pSF-04, was digested with the *Mfe*l restriction enzyme to excise the insert bearing the lacZ encoding sequence. pSF-04 was then purified on agarose gel using the NucleoSpin Extract kit (Macherey Nagel) and treated with T4 DNA polymerase in the presence of 2.5 mM of dGTP for 30 min at 22°C before inactivating the enzyme by heating 20 min. at 75°C.

The cloning step of the two fungi TPK genes consisted in a hybridization reaction performed by mixing 0.01 pmol of pSF-04 and 0.02 pmol of the insert in a reaction volume of 3 μl and an incubation time of 5 min. at 22°C, 1 μl of 25 mM EDTA was then added to the reaction mixture. After a second incubation period of 5 min. at 22°C, the entire hybridization reaction was used to transform *E. coli *DH5α. Selection was performed on LB plates containing 100 μg/ml ampicillin, positive plasmids were then isolated. This cloning procedure allowed insertion of the gene of interest in frame with a sequence encoding the N-terminal (His)_6 _tag and a GHHHQL sequence corresponding to the translation of the forward primer sequence. An additional C-terminal fragment QLDGDLEAA corresponds to the translation of a linker between the gene of interest and the sequence encoding the GFP.

An expression screening was performed using our standard procedure [11] and the GFP gene reporter was used to quantify the soluble expression through fluorescence measurements [12] in order to identify the best condition for the fungi TPK proteins soluble expression. Subsequent removal of the GFP encoding gene was achieved by digesting the plasmid by *Not*I followed by intra ligation.

As a result, the plasmids carrying the CA1462 gene were over-expressed in *E. coli *Origami in 1L flasks containing SB medium cultured overnight at 17°C. Induction was performed using IPTG (500 μM) when the culture reached an OD_600 nm _of 0.5. The plasmids carrying the YOR143C gene were expressed in *E. coli *Origami in 1L flasks containing 2YTG medium at 25°C after induction with IPTG (500 μM) when the culture reached an OD_600 nm _of 0.5. After centrifugation, the pellets were resuspended in buffer A (50 mM NaH_2_PO_4_, 300 mM NaCl pH 8.0) with 5 % glycerol and 0.1 % Triton X-100 then sonicated and centrifuged again.

### Purification

The cleared lysate was applied to a 5 ml HiTrap Chelating Column (GE Healthcare) loaded with Ni^2+ ^and equilibrated with buffer A. The column was washed with 10 column volumes of buffer A, 10 column volumes of buffer A containing 25 mM Imidazole and 5 column volumes of buffer A containing 50 mM Imidazole at a flow rate of 1 ml.min^-1^. Elution was performed with a linear gradient over 7 column volumes from 50 mM to 500 mM Imidazole. The fractions corresponding to the elution of CA1462 and YOR143 TPK proteins with 150–200 mM Imidazole were run on a desalting column (Fast Desalting Column HR 10/10, Pharmacia) and analyzed by mass spectroscopy and N-terminal Edman sequencing. After purification, the fractions contained at least 98% pure protein in Tris buffer 10 mM pH 8 (CA1462) and Tris buffer 20 mM, 100 mM NaCl pH 8 (YOR143). For the CA1462 protein, the isoelectrofocalisation revealed a band around 6.0 instead of the predicted pI of 5.4. Gel filtration of the purified TPK proteins on a Sephacryl S200 HR column indicated the final products were dimeric in solution.

### Enzymatic assays

In order to verify that our two *C. albicans *and *S. cerevisiae *enzymes were active TPK we developed a protocol involving four consecutive reactions and different enzymes. The TPK activities were determined at room temperature using purified protein (10 μg.ml^-1^) in Tris buffer (50 mM, pH 7.5) in presence of 1 mM thiamine and 5 mM ATP. The reaction cascade was monitored through the NADH decrease in absorbance at λ = 334 nm. The first reaction is catalyzed by the TPK and produces a TPP and an AMP. The myokinase then uses the produced AMP in the presence of ATP to produce 2 ADP molecules, each of them being used by the pyruvate kinase enzyme in presence of phosphoenol pyruvate (PEP) to produce a pyruvate and an ATP molecule. The last step is catalyzed by the lactate dehydrogenase hydrolyzing pyruvate in the presence of NADH to produce L-lactate and NAD^+^.

### Crystallisation

The *C. albicans *TPK recombinant protein was concentrated to 20.4 g/L in 10 mM Tris buffer at pH 8.0 using a centrifugal filter device (Ultrafree Biomax 30 K, Millipore, Bedford MA, USA). Precipitation experiments were carried out at 293 K using various precipitating agents (i.e. AmSO_4_, PEG, NaCl, MPD) at various pHs (i.e. 5, 6, 7, 8, 9) to determine the optimal protein concentration for crystallization. The screening for crystallization conditions was performed on 3 × 96-well crystallization plates (Greiner) loaded by an 8-needle dispensing robot (Tecan, WS 100/8 workstation modified for our needs), using a 1 μl sitting drop per condition at 293 K. The tested 576 crystallization conditions include in-house designed [13] and commercially available solution sets (MDL Structure-Screen, Wizard-Emerald BioSystems).

The TPK protein (10.2 g.l^-1^, buffer Tris 10 mM, pH 8.0) was then incubated with AMP-PNP (1 mM) and/or thiamine (5 mM) for few minutes and the best crystals of each protein/ligand complex were obtained using the hanging drop vapor diffusion method with a 1 ml reservoir. Crystallization droplets were made of 0.5 μl of complex mixed with 0.5 μl of the reservoir solution made of 17.5 to 20 % PEG4000, MgCl_2 _0.2 M, Tris 0.1 M, 20 % Glycerol between pH 7.0 and 7.5. Crystals appeared within a few days.

### Data collection

Crystals of the TPK protein were collected in a Hampton Research 0.2 mm^3 ^loop, flash frozen to 100 K in a cold nitrogen gas stream and subjected to X-rays. A first data set was collected from a crystal of TPK incubated with thiamine on a MarCCD (165 mm) camera at the European Synchrotron Radiation Facility (ESRF) on the BM30A-FIP beamline at a wavelength of 0.954 Å. A second data set corresponding to a crystal of TPK incubated both with thiamine and AMP-PNP was collected at ESRF on ID29 beamline on an ADSC Q210 2D detector at a wavelength of 0.98 Å. The diffraction data were indexed with MOSFLM [14] and scaled with the SCALA [15] software from the CCP4 suite [16]. The two crystals belong to the space group P_1 _with two molecules in the asymmetric unit. All statistics of the processed data are summarized in table 1.

**Table 1:** X-ray data collection (ESRF) and refinement statistics

**Data collection**	**TPK Thiamine-PNP**	**TPK Thiamine**
Beam line	ID29	BM30A
Wavelength (Å)	0.98	0.954
Space group	P1	P1
Unit cell (Å)	a = 50.825 b = 60.331 c = 63.722	a = 51.309 b = 60.696 c = 64.831
100 K	α = 66.144 β = 89.941 γ = 65.067	α = 65.937 β = 89.858 γ = 64.868
Resolution range (Å)(highest resolution shell)	29.45 to 1.96(2.03 to 1.96)	50.19 to 2.1(2.21 to 2.1)
Observations	70690 (104)	68171 (8700)
Unique reflections	35345 (52)	33349 (4258)
Multiplicity^1^	1.9 (1.0)	2 (2)
Completeness^1^	92.8 (60.3)	96.1 (85.4)
<I/σI>^1,2^	14.5 (3.8)	7.9 (7.1)
R_sym _(%)^1,3^	3.5 (19.5)	4.7 (8.3)

**Refinement**	**2G9Z**	**2HH9**

R_cryst _(%)^4^	18.7 (31)	18.1 (21)
R_free _(%)	23.6 (33)	25.4 (26)
Δ_bond _(Å)	0.022	0.05
Δ_angle _(°)	1.934	1.2
N° Protein atoms	4776	4786
N° water	520	660
N° Heterogen atoms	62	40
Average B factor (Å^2^)	26.9	26.2
Protein main chain	26	25.9
Water	41	45.8
Ligand	18.1	20.4
Mg^2+^	23.58	8.4
PO_4_	31.6	
Cl^-^	29.8	
Ramachandran plot (%)		
Most favored	79.2	76.0
Allowed	9.0	11.8
Generously allowed	0.3	0.3
Disallowed regions	0.3	0.3

^1 ^values in parentheses are for the highest resolution shell.

^2 ^< I/σI>, is the mean signal to noise ratio, where I is the integrated intensity of a measured reflection and σ is the estimated error in the measurement.

^3 ^*R*_*sym *_= ∑_*h *_∑_*i*_|*I*_*h, i *_- ⟨*I*_*h*_⟩|/∑_*h *_∑_*i*_|*I*_*h, i*_, where I is the integrated intensity of reflection h having i observations and ⟨*I*_*h*_⟩ is the mean recorded intensity of reflection h over multiple recording.

^4 ^R_cryst _= ∑||*F*_*o*_|-|*F*_*c*_||/∑|*F*_*o*_|, where F_o _are observed and F_c _calculated structure factor amplitudes. R_free _is calculated from a randomly chosen 9.9% of reflections.

### Structure determination

The *C. albicans *TPK crystal structure in complex with thiamine has been determined by molecular replacement using the CaspR server [17,18]. To generate the *C. albicans *TPK models, we used the available three-dimensional structures of *Saccharomyces cerevisiae *and *Mus musculus *thiamine pyrophosphokinase (PDB id 1IG0 and 1IG3) as template and TPK related sequences from other species (Swiss-Prot id Q9H3S4, P41888). MODELLER produced 15 models which were screened for a molecular replacement solution using 15 to 3 Å data and the AMoRe software [19]. The space group was first misinterpreted as C_2 _with one monomer per asymmetric unit and cell parameters of a = 109.15 b = 50.83 c = 63.81, β = 116.18. The structure was solved in this space group. The best model produced a solution with one monomer and a correlation of 29.3% and a R-factor of 53.7% prior to CNS refinement. After one round of rigid-body refinement and minimization using the CNS program [20] with 15 to 3 Å data, the R-factor dropped to 42% with a 50% R_free_. Rapidly, the refinement stopped converging and we suspected wrong symmetry assignation. We thus reprocessed the TPK/thiamine/AMP-PNP data in P_1 _space group and computed a self-rotation function using AMoRe which resulted in a peak corresponding to a two-fold pseudo symmetry with a correlation coefficient of 93.4% using data between 15 and 2 Å thus confirming the P_1 _space group. The two monomers, related by a non crystallographic symmetry (NCS), mainly present differences in flexible loops and water molecules. The model was manually corrected using TURBO-FRODO [21] followed by several rounds of minimization and individual B-factors refinements using 29.45 to 1.96 Å data and the CNS software. 520 water molecules were built into the model as well as a thiamine-PNP molecule, three Mg^2+ ^and one Cl^- ^ion per monomer. Some residues were still disordered in loops L_1_, L_2 _and L_4 _and are absent from the deposited structure. The final free and working R-values are 23.6% and 18.7% respectively.

The refined structure was then used against the TPK/thiamine data and refinement was performed using several manual rebuilding cycles followed by minimization and B-factor refinement using COOT [22] and REFMAC [23]. 660 water molecules were built into this model as well as a thiamine molecule and two Mg^2+ ^ions per monomer. Again, some residues were still disordered in loops L_1_, L_2_, L_4 _and L_11 _and are absent from the deposited structure. The final free and working R-values are 25.4% and 18.1% respectively.

## Results

### TPK activity of the *C. albicans *recombinant protein

The activity of the *S. cerevisiae *TPK protein was previously measured on the native protein extracted from yeast [2]. To validate our indirect enzymatic assay, we used the *S. cerevisiae *enzyme as a TPK activity control. Under the conditions described in the methods section we measured specific activities of 0.266 nM/min/mg for the *S. cerevisiae *enzyme (compared to 0.27 nM/min/mg for the native enzyme [2]) and of 0.367 nM/min/mg for the *C. albicans *recombinant protein.

### The CA1462 overall structure: monomer

**Figure 1:**
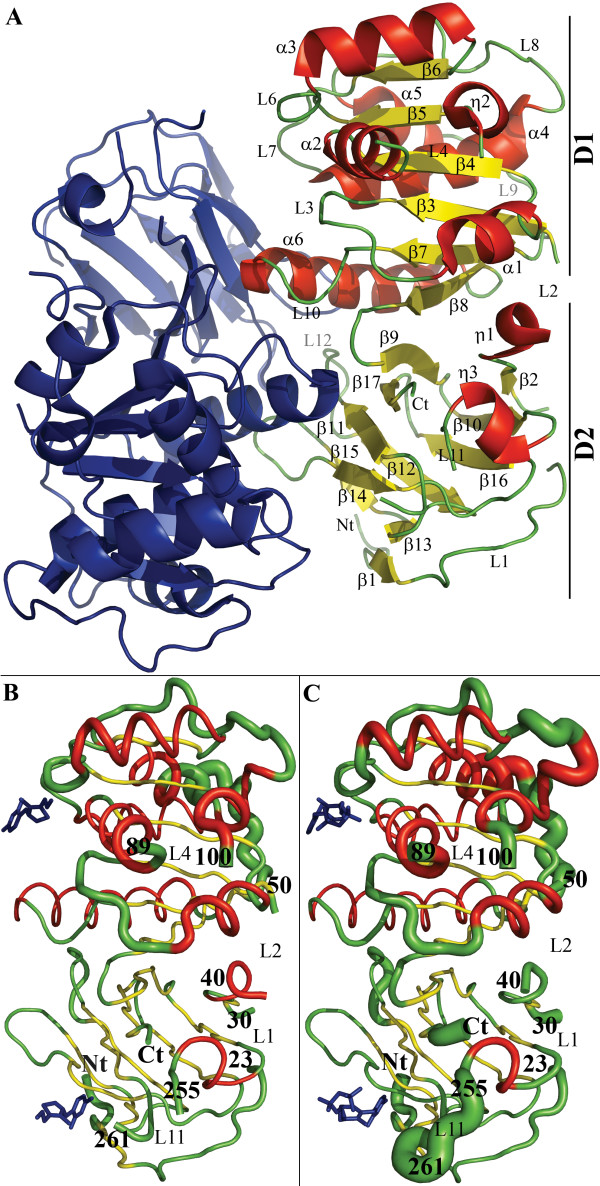
**Overall structure of the *C. albicans *TPK enzyme**. A) The first monomer is represented in blue and the second monomer is in yellow for β-sheets, in red for helices and in green for loops. D1 corresponds to the αβ domain and D2 to the β-sandwich domain. Representation of the B-Factor values on the structure of *C. albicans *TPK in complex with thiamine (B) or Thiamine-PNP (C). The tube radius is correlated with B-factors values (high, large tube and small, thin tube). Structures are color coded according to secondary structure elements (red for helices and yellow for β-strands). Ligands are represented in blue. All the structure representations were generated using PYMOL [34].

As expected, the overall structure of the *Candida albicans *TPK is similar to other members of the Thiamine pyrophosphokinase family. CA1462 is a homodimer, with each monomer constituted of two domains. The D1 domain (Figure 1A, see Additional file 2) is an αβ fold with a twisted β-sheet made of six parallel strands (β _3 _to β _8_) surrounded by six helices (α_1_1 to α_6_). The D2 domain is a β sandwich composed of two layers arranged in a jelly-roll topology (Figure 1A, see Additional file 2). Antiparallel strands β _1_, β _10_, β _12_, β _13_-β _14_, β _16_, and β _2 _parallel to β_10 _constitute the first layer. Antiparallel strands β _9_, β _11_, β _15 _and β _17 _constitute the second one. Both domains contribute to the dimeric association with a 1920 Å^2 ^buried surface area.

**Figure 2:**
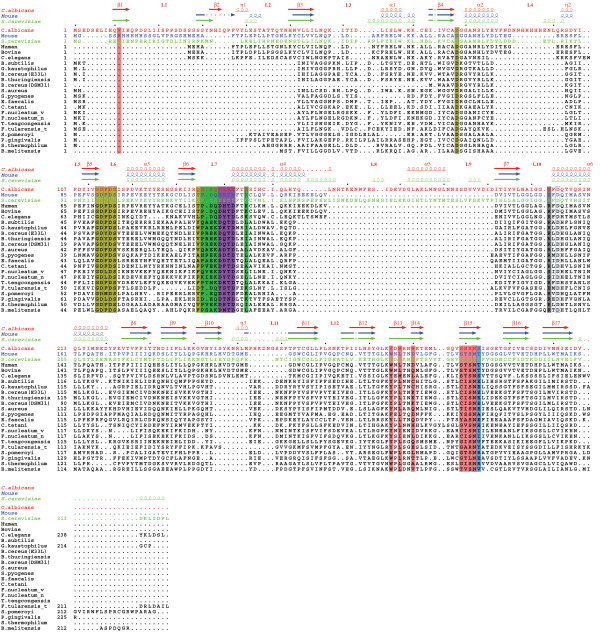
**Multiple structural alignment of TPK sequences**. Mouse in blue (PDB id 2F17), *Candida albicans *SC5314 in red (Swiss-Prot id CA1462), *Saccharomyces cerevisiae *in green (PDB id 1IG0), Human (Swiss-Prot id Q9H3S4), Bovine (Swiss-Prot id Q5E9T4), *Caenorhabditis elegans *(Swiss-Prot id P30636), *Bacillus subtilis *(Swiss-Prot id O34664], *Geobacillus kaustophilus *(Swiss-Prot id Q5L0R6), *Bacillus cereus *(strain ZK/E33L) (Swiss-Prot id Q636G7), *Bacillus thuringiensis *subsp. *Konkukian *(Swiss-Prot id Q6HEV6), *Bacillus cereus *(strain ATCC 14579/DSM 31) (Swiss-Prot id Q819U8), *Staphylococcus aureus *(strain COL) (Swiss-Prot id Q5HGL0), *Streptococcus pyogenes *serotype M6 (Swiss-Prot id Q5XDX1),*Enterococcus faecalis *(Swiss-Prot id Q82ZE3), *Clostridium tetani *(Swiss-Prot id Q895P3), *Fusobacterium nucleatum *subsp. *vincentii *ATCC 49256 (Swiss-Prot id Q7P831), *Fusobacterium nucleatum *subsp. *nucleatum *(Swiss-Prot id Q8RF31), *Thermoanaerobacter tengcongensis *(Swiss-Prot id Q8R9T9), *Francisella tularensis *subsp. *Tularensis *(Swiss-Prot id Q5NFC3), *Silicibacter pomeroyi *(Swiss-Prot id Q5LTU1), *Porphyromonas gingivalis *(Swiss-Prot id Q7MTP9), *Symbiobacterium thermophilum *(Swiss-Prot id Q67QK6), *Brucella melitensis *(Swiss-Prot id Q8YJ01). Secondary structures are numbered as in *C. albicans*. Strictly conserved residues are boxed with a thin blue line. *C. albicans *TPK residues making contacts with Mg^2+ ^ions are highlighted in brown, those in contact with phosphate and Mg^2+ ^in green, those in contact with thiamine or thiamine-PNP in pink, and those in contact with thiamine or thiamine-PNP and Mg^2+ ^in purple. The residues exclusively interacting with thiamine-PNP (and not thiamine) are in grey, and those in contact both with thiamine-PNP and phosphate are in light blue.

We used the 3D-COFFEE server [24,25] to perform a structural alignment of 23 selected TPK sequences of various origins using CA1462, *Saccharomyces cerevisiae *(PDB id 1IG0) [8] and *Mus musculus *(PDB id 2F17) [7] TPK as structural templates. Despite the high variability among these TPK sequences (20% to 45% identity with CA1462), there is strong residue conservation around the catalytic center (Figure 2, see Additional file 3). Four aspartates are strictly conserved (D78, D113, D115, D142, according to the *C. albicans *TPK numbering) and have already been proposed to be involved in the stabilization of the ATP molecule through Mg^2+ ^contacts [8]. An aromatic residue is always found at position 285 (Y285 in *C. albicans*) and is known to participate in the thiamine stabilization [26]. An asparagine residue (N302) is strictly conserved but its function is not yet understood. This residue is close enough to the thiamine substrate to make some contacts with its amidopyrimidine ring.

Interestingly, there are three loops in the *C. albicans *TPK structure. Two of them, L_4 _and L_11 _(Figure 2), are unique to *C. albicans*, while the third and longest insert (L_8_-α_5_-L_9_) is also present in the yeast homolog with which it shares less than 20% identity over 35 residues (Figure 2). None of these fragments appear to be directly involved in the catalytic reaction or in the ligands binding (Figure 2). In the structure, the L_11 _loop lies in the solvent, in an open conformation and away from the rest of the molecule (Figure 1B–C). The B-factors for this loop are also very high (see Additional file 4). We first built an approximate loop by following the residual density present in the 2Fo-Fc and Fo-Fc maps. We then used manual docking to verify that the length and geometry of this loop was compatible with a transient interaction with the ligands. This loop may thus play a role during the binding of the substrates, or in the pyrophosphorylation mechanism itself.

### The CA1462 overall structure: dimer

The main differences between the two monomers related by the NCS involve flexible loops disordered in the crystal structure (Figure 1B–C, see Additional file 4). They include loops L_1_, L_2 _and L_4_, which are too distant to be involved in the active site formation and located too far from symmetry related molecules to possibly be directly involved in the crystal packing. There are also water molecules which are not strictly symmetrical between the two monomers (data not shown). Although the loop L_11 _is highly mobile and associated with high B-factors (Figure 1B-C, see Additional file 4) it presents little differences between the two monomers. The L_8_-α_5_-L_9 _fragment, which is involved in crystal packing through interactions between L_8_-α_5 _helix/turn and α_3_-β_6 _helix/turn in the two monomers, can be superposed with less than 0.3 Å root mean square deviation (RMSD) based on α carbon (Cα) superposition.

There are no major differences between the TPK known structures based on Cα superposition using LSQMAN [27]. RMSD are 2.33 Å (534 superimposed residues) between the *C. albicans *and the *S. cerevisiae *TPK structures, and 2.62 Å (464 superimposed residues) between the *C. albicans *and the Mouse TPK. The backbone trace around the thiamine binding site superimposes well except for one fragment. Timm *et al*. described a rearrangement of the L_6 _loop in the mouse TPK when comparing the structures with thiamine and TPP [7]. This rearrangement is not observed within the *C. albicans *TPK which fits the mouse TPK in complex with TPP except for the S116 side chain.

### Thiamine binding site (PDB id 2HH9)

**Figure 3:**
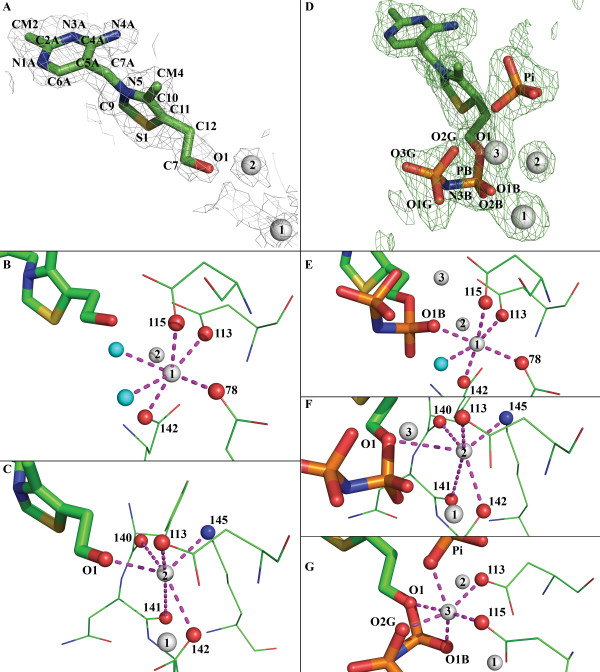
**Ligands electronic density map and Mg^2+ ^coordination**. 2fo-fc electronic density map of thiamine contoured at 1 sigma (A) and coordination of each Mg^2+ ^ion in the TPK/thiamine complex (B-C). Fo-Fc electronic density map contoured at 1.5 sigma (displayed in green) of TPNP (D) with Mg^2+ ^and PO4^3-^. This map was computed using the refined structure where all ligands have been removed. Coordination of each Mg^2+ ^ion in the TPK/thiamine-PNP complex (E-G). Magnesium are represented as white spheres, water molecules as cyan spheres, oxygen atoms are in red, nitrogen in blue and carbon in green. Mg^2+ ^coordinations are marked with magenta dashed lines.

Two binding sites can be shown in the asymmetric unit. The strong electronic density is consistent with thiamine molecules present at the thiamine binding site (Figure 3A). As in the mouse structure, the thiamine moieties are located at an extremity of the groove formed by the dimer assembly. For convenience, we will take A as the reference monomer and B as the second one (according to the deposited PDB structures) but all observations made on one of the active sites can be transposed to the second one. The ligands are maintained by both main chains and side chains interactions. These interactions involve the strands β_13_, β_14_, β_15_, of monomer B and the loops L_6_, L_7 _of monomer A.

**Figure 4:**
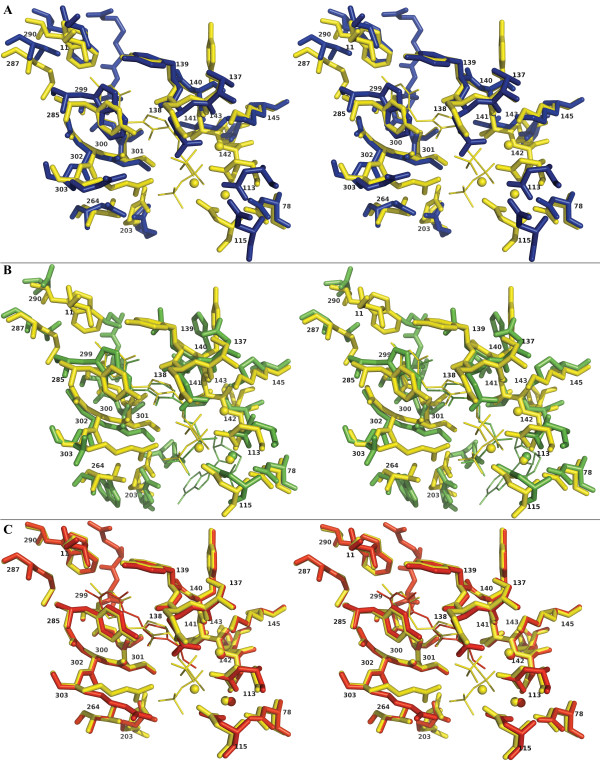
**Stereo view of the superposed ligand binding sites from different species**. The superposition was obtained using as reference structure (in yellow) the *C. albicans *TPK co-crystallised with TPNP (PDB id 2G9Z) and A) *S. cerevisiae *(Blue) (PDB id 1IG0), B) Mouse (green) (PDB id 2F17) C) *C. albicans *TPK co-crystallised with thiamine (red) (PDB id 2HH9). Residue numbers correspond to *C. albicans *numbering.

As observed in other structures (PDB id 2G9Z, 1IG3 and 2F17) there is a weak stacking between an aromatic residue (Y285B in CA1462) and the amidopyrimidine ring (Figure 4A–C). The CM2 carbon makes hydrophobic interactions with the W290B side chain, a residue conserved in the sequence of the *S. cerevisiae *homologue. In the mouse sequence this residue is replaced by an aspartate and does not make any contact with the ligand. Nevertheless, a certain hydrophobic environment is maintained in the murine structure with the spatial proximity of a leucine (L204). The thiamine ammonium group N4 of the amidopyrimidine ring is engaged in a salt bridge with the Q138A main-chain carbonyl oxygen (Figure 4A). The opposite side of this ring is stabilized by another salt bridge involving N1 and the oxygen from the S299B side chain (Figure 4A). The aspartate residue interacting with N4 and N3 described in the mouse structures [26] is replaced by a tyrosine (Y139B) too far away from the ring to interact with it. N5 from the thiazolium ring is stabilized by electrostatic interactions between the main chain carbonyl oxygen atoms of S300B and Q138A.

At least 2 residues involved in the thiamine binding pocket (Y139A, W290B) are poorly conserved between different species but well represented in fungus (alignment not shown) and could be the focus of specific antifungal drug design.

### Magnesium binding site in the TPK/Thiamine complex

As observed in the mouse TPK/TPP complex, an Mg^2+ ^ion is maintained by electrostatic interactions with the side chain of the four strictly conserved aspartate residues (D78, D113A, D115, D142). The 2 last coordinations are made with water molecules 92 and 69 (Figure 3B, Mg^2+ ^number 1).

Timm *et al*. highlighted that a second Mg^2+ ^ion could be involved in the reaction process as in another pyrophosphokinase (HPPK). Interestingly, the *C. albicans *TPK/thiamine complex reveals a second Mg^2+ ^partially stabilized by the hydroxyl group O1 of thiamine and a salt bridge with D113 and D142 side chains and with two main chain carboxy oxygens (Y140, N141). The nitrogen atom from the K145 side chain participates in the last coordination (Figure 3C, Mg^2+ ^number 2). As proposed by Timm *et al*., this lysine could be involved in the activation of the thiamine hydroxyl by the stabilization of a water molecule [7] or, as in this *C. albicans *structure, of a magnesium ion.

### Thiamine-PNP binding site, implications for the ATP binding site (PDB id 2G9Z)

In order to compare the *C. albicans *and mouse TPK binding sites, we used 2 ATP analogues (AMP-CPP and AMP-PNP) during the crystallization process. In the AMP-CPP molecule a carbon atom replaces the oxygen atom located between the α and β phosphates in the ATP molecule (O3). In the AMP-PNP molecule, a nitrogen atom (N3) replaces O3. While we did not get crystals of the TPK/Thiamine/AMP-CPP complex, the TPK incubated with thiamine and AMP-PNP produced usable crystals. However, the structure revealed that instead of a TPK/Thiamine/AMP-PNP complex, we obtained an unexpected TPK/thiamine-PNP complex with a TPNP molecule in the enzyme binding site (Figure 3D). This result is consistent with the earlier suggestion that the pyrophosphate group is transferred at once from the ATP molecule to the thiamine moiety.

The superimposition of the *C. albicans *TPK/TPNP complex onto the mouse TPK/TPP complex (PDB id 2F17) highlights few differences between the two thiamine phosphate ends. The nitrogen N3 in the TPNP molecule is symmetrically opposed to its equivalent oxygen O3 in the TPP molecule. It is stabilized by a weak electrostatic interaction (3.28 Å) with the S301B γ-oxygen in the *C. albicans *TPK/TPNP complex (Figure 4). In the mouse TPK/TPP complex, this serine residue, located in the AMP binding pocket, is at 4.44 Å from the O3. We propose that this serine residue could repulse the TPP in normal conditions and help to release it out of the active site. In the Mouse TPK/TPP complex, Q134A interacts directly with the phosphate end of the thiamine. The equivalent glutamine (Q206A) adopts the same conformation in the *C. albicans *TPK/thiamine complex and an opened conformation in the *C. albicans *TPK/TPNP complex.

In the mouse TPK complex with AMP and TPP [7], the ATP binding site involves residues poorly conserved across different species. In the *C. albicans *TPK structure, no extra density that could correspond to a nucleotide was found even if this site is opened enough to accommodate an ATP molecule.

**Figure 5:**
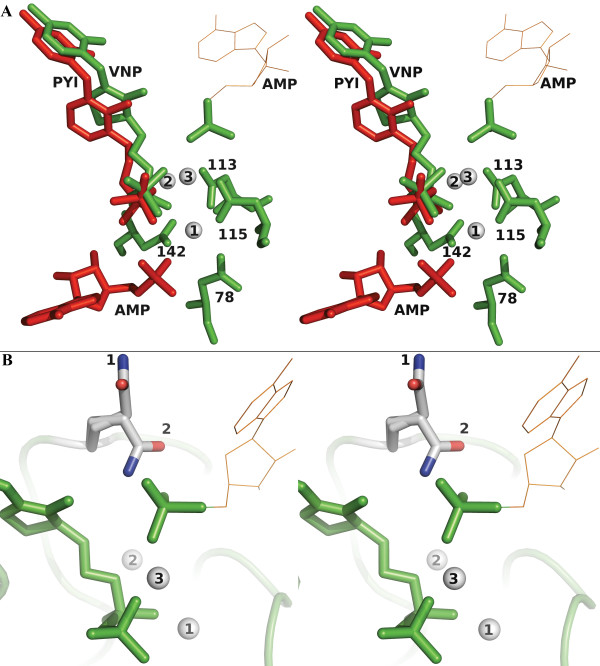
**AMP binding site**. A) Stereo view of the binding site symmetry in the *C. albicans *TPK (green) superposed on the mouse TPK (red). Hypothetical AMP is represented with brown lines. B) Stereo view of the 2 alternative conformations of Q138A (gatekeeper) in grey with TPNP (green), Mg^2+ ^(white) and AMP (brown lines) in the potential secondary binding site.

Interestingly, exactly opposed to the mouse AMP binding site, (Figure 5A, see Additional file 3), we have identified an inorganic phosphate (Pi) (average B-factor of 30 and occupancy of 1). This phosphate is stabilized by a Glutamine (Q138A), an Arginine (R303B) and a salt bridge involving a magnesium ion (described later). Moreover, despite the lack of strong residual density, an AMP molecule could reasonably be accommodated at that position with an average B-factor of 50 and a good occupancy (0.8). However, the non-phosphate part of this putative AMP molecule presents higher B-factors (around 60) and interacts with side chains of poorly conserved residues (see Additional file 3): R134A, Q138A and S136A directly or through a water molecule bridge. It also interacts with the carbonyl oxygen of D113A.

In the *C. albicans *thiamine complex, 2 water molecules take the place of oxygen atoms from the proximal thiamine phosphate. Similarly to the *S. cerevisiae *apo form, a third water molecule occupies the same position as the distal thiamine phosphorus atom in the Thiamine-PNP complex.

The side chain of the Q138 can adopt two alternative conformations: the one observed in *S. cerevisiae *or mouse TPK (PDB id 1IG0, 2F17) and the one observed uniquely in the *C. albicans *TPK/TPNP complex (Figure 5B). In the latter conformation, Q138 does not interact with the inorganic phosphate described above.

### Magnesium binding site in the TPK/TPNP complex

Three magnesium ions are stabilized in the active site. The first two ions superimpose exactly with the ones co-crystallised in the *C. albicans *TPK structure with thiamine. One is coordinated through the δ-oxygen of each of the four conserved aspartates and the oxygen of the proximal phosphate in TPNP. The sixth Mg^2+ ^coordination involves a water molecule in *C. albicans *TPK instead of the AMP-phosphoryl in the mouse structure (Figure 3E, Mg^2+ ^number 1). The second one is stabilized by the same interactions as in the thiamine/TPK complex except for the O1 atom of the thiamine which is involved in a thiamine-PNP ester bond rather than an hydroxyl group in thiamine (Figure 3F, Mg^2+ ^number 2).

The third magnesium ion is coordinated by the δ-oxygen from D113 and D115 and one oxygen atom from each phosphate group of the thiamine-PNP. The last coordination of this Mg^2+ ^involves an oxygen atom from the free inorganic phosphate symmetrically positioned with regard to the AMP phosphate shown in the mouse ATP binding site (Figure 3G, Mg^2+ ^number 3).

## Discussion

### Identification of a potential secondary binding site

Timm *et al*. described the 3D structure of the mouse thiamine pyrophosphokinase co-crystallized with AMP, pyrithiamine pyrophosphate (PPP) and magnesium ions [7]. Their results confirmed that the AMP (and maybe the ATP) is maintained in the protein by interactions with residues 57, 58, 77 to 79, 141 to 143, 199 to 203, 206 and 207. Although we did not obtain any nucleotide analogue in our structures along with the TPNP and magnesium ions, we have identified an inorganic phosphate in the structure located differently to the AMP molecule in the mouse TPK structure. This phosphate is diametrically opposed to the AMP phosphoryl group described by Timm *et al*. relative to the thiamine moiety (Figure 5A) and can have two distinct origins. First, AMP-PNP is known to be very unstable in acidic conditions and can hydrolyze into an equivalent phosphoramidate and inorganic phosphate (Sigma report [28]). Our crystals have been obtained at pH 7.0 with a large excess of AMP-PNP, but a partial degradation of AMP-PNP cannot be excluded. Second, this phosphate may originate from the AMP used in the pyrophosphorylation of the thiamine. The co-crystallized inorganic phosphate highlights a very high symmetry in the active site (Figure 5A). The residues involved in this phosphate stabilization are poorly conserved across the different known TPKs (Figure 2, see Additional file 3). The stabilization of the non-phosphate part of the AMP in this alternative site seems to be restricted to the ribose part of the nucleotide, which suggests the existence of a rather non specific binding site which could accommodate nucleotides other than ATP, namely GTP, UTP and TTP [29]. Alternatively, it could correspond to a transitional site for the AMP on its path out of the protein, or for the ATP on its way to the active site, and thus guide the motion of this product/substrate during the hydrolysis process.

### Existence of a gatekeeper residue?

Mutational studies of the human TPK, sharing 29 % identity over 288 amino-acids with the C. albicans TPK, showed that residues D71, D73 and D100 (corresponding to D113A, D115A and D142A in C. albicans) play a crucial role in carrying out the catalytic process, that Q96 and D133 (corresponding to Q138A and D205A) are involved in the binding of thiamine, and that T99 and R131 (corresponding to N141A and R203A) interact with the ATP [29]. In our structures, Q138A adopts two main conformations: one opened and one closed with respect to the active site access. The structure containing thiamine only exhibits the closed conformation, while both are present in the TPK/TPNP complex. As in other kinase structures, Q138A could play the role of a gatekeeper, one of the major determinants of the selectivity in these proteins [30,31]. If a secondary binding site exists in the protein, this residue could directly interact with the phosphoryl group of the AMP and both the ribosyl and pyrimidine part of the nucleotide (Figure 5B), thus playing a role in the enzyme selectivity. In the event of a transitional site, Q138A could modulate the release of AMP or the entrance of ATP.

### Hypothesis on the pyrophosphorylation mechanism in *C. albicans *TPK

The mechanism of pyrophosphorylation is not well understood. Structural studies complemented by enzymatic assays and mutation experiments, could give us insight into this process conserved in a lot of species.

Baker *et al*. [8] proposed that, in yeast TPK, the three aspartates corresponding to D78A, D113A, A142A could play the same role as in the HPPK (6-Hydroxymethyl-7,8-dihydroprotein pyrophosphokinase). In this enzyme, the pyrophosphate (PPi) is transferred from an ATP to an HP (6-Hydroxymethyl-7,8-dihydroprotein) through a one step pyrophosphorylation mechanism. The PPi is maintained in the proper conformation by two aspartates through the coordination of two Mg^2+ ^ions [32].

In an attempt to co-crystallize TPK with its two substrates or in an intermediate state, we used AMP-PNP and AMP-CPP, two ATP analogues. In the AMP-PNP, the bond between β and γ phosphates is non-hydrolysable, excepted under acidic conditions, whereas in AMP-CPP it is the bond between α and β phosphates which is non-hydrolysable. Our attempts to co-crystallize AMP-CPP or AMP-PNP with the enzyme have failed. Nevertheless, using AMP-PNP, as suggested by the electronic density maps, the PNP group was transferred to the thiamine to form a thiamine-PNP. This observation is consistent with a one step PPi transfer. A steady-state kinetics study of the human TPK led to the proposal that the mechanism is a ping-pong reaction [29] as proposed for yeast [33]. According to our results, and in agreement with Timm *et al*., the possibility to co-crystallize the two substrates at the same time is inconsistent with an ordered ping-pong mechanism where the thiamine binds the enzyme after ATP has been hydrolyzed and the AMP released. Moreover, the presence and position of a possible secondary AMP binding site is more likely to support a mechanism where the AMP is released after the PPi transfer and before the TPP molecule.

To our knowledge, this study is the first to report more than one magnesium ion in the TPK active site with one or two additional Mg^2+ ^properly positioned to participate in the pyrophosphorylation process in a mechanism akin to the one proposed by Timm *et al*. [7]. Where Timm *et al*. showed a water molecule, our structures exhibit a magnesium ion at a strategic location. After the binding of an ATP molecule, K145A, a strictly conserved lysine, could activate the thiamine hydroxyl extremity through the coordination of the first Mg^2+^. At the same time, the 4 aspartates (78A, 112A, 115A and 142A) could activate the PPi moiety of the ATP molecule. The transfer could then take place. Each *C. albicans *structure reveals new positions suggesting that 3 Mg^2+ ^ions could be involved in the PPi transfer mechanism. The third Mg^2+ ^could stabilize the PPi during the transfer.

## Conclusion

In summary, we have determined the X-ray structure of the *C. albicans *thiamine pyrophosphokinase co-crystallized with the thiamine or the thiamine-PNP and an inorganic phosphate. These different structures allow us to describe several original features. The active site symmetry and the inorganic phosphate binding site suggest the presence of an alternative binding site in *C. albicans *TPK, or at least a transitional position of the substrate/product along the reaction pathway. This also suggests an enzymatic process where both products, TPP and AMP, are present at the same time in the protein with the release of the AMP molecule preceding the release of the TPP. We propose that the Q138A could interact with both substrates and products and work as a gatekeeper, modulating the activity of the enzyme (as observed in other kinases). The crystallization of thiamine-PNP at the TPK active site strongly suggests that the pyrophosphorylation process is a one step mechanism. K145A seems to be important in the catalytic process and substrate activation. The presence of two or three magnesium ions at the same time in the active site in these structures suggests a catalytic reaction similar to the one described for HPPK, also performing pyrophosphate transfer. The PPi transfer mechanism at work in thiamine pyrophosphokinases could be further explored by studies focusing on mimicking the transition state.

## Abbreviations

TPK: thiamine pyrophosphokinase; TPP: thiamine pyrophosphate; PPi: Pyrophosphate; TPNP: thiamine-PNP; AMP-PNP: Adenosine 5'-(β,γ-imido)triphosphate; PNP: (β,γ-imido)diphosphate; HPPK: 6-Hydroxymethyl-7, 8-dihydropterin pyrophosphokinase; LIC: ligation-independent cloning; NCS: Non Crystallographic Symmetry.

## Authors' contributions

SS performed research, analyzed data and wrote the paper. VM, NM and TR designed research and contributed to new reagents/analytic tools. CS: performed research. J–MC: designed research and wrote the paper. CA designed research, performed research, analyzed data and wrote the paper. All authors read and approved the final manuscript.

## Footnotes

The atomic coordinates and structure factors for the crystal structures of the thiamine pyrophosphokinase from *Candida albicans *in complex with thiamine or thiamine-PNP are available in the RCSB Protein Data Bank under PDB id 2HH9 and 2G9Z respectively.

## Supplementary Material

Additional file 1 pSF-04 expression vector map. Click here for file

Additional file 2 **Stereo ribbon diagram of the *C. albicans *TPK monomer structure **color coded according to secondary structure elements (helices in red, β-sheet in yellow and loops in green). Click here for file

Additional file 3 **Molecular surface representation of the *C. albicans *TPK active site**. Conserved residues in contact with at least one ligand are colored in blue and variable ones in red. All other residues are colored in grey. The ligands are colored with the Pymol default colors and Mg^2+ ^ions are in white. Click here for file

Additional file 4 **Graph representation of the B-factors values of the *C. albicans *TPK structures **in complex with thiamine and thiamine-PNP. Monomers A and B of TPK/Thiamine-PNP are shown in black and grey respectively. Monomers A and B of TPK/Tiamine are shown in red and orange respectively. Click here for file
