# Supplementary figures and images for: Structural characterization of CA1462, the Candida albicans thiamine pyrophosphokinase
Source: BMC Struct Biol. 2008 Jul 24;8:33. doi: 10.1186/1472-6807-8-33 (PMC2515308; doi:10.1186/1472-6807-8-33)

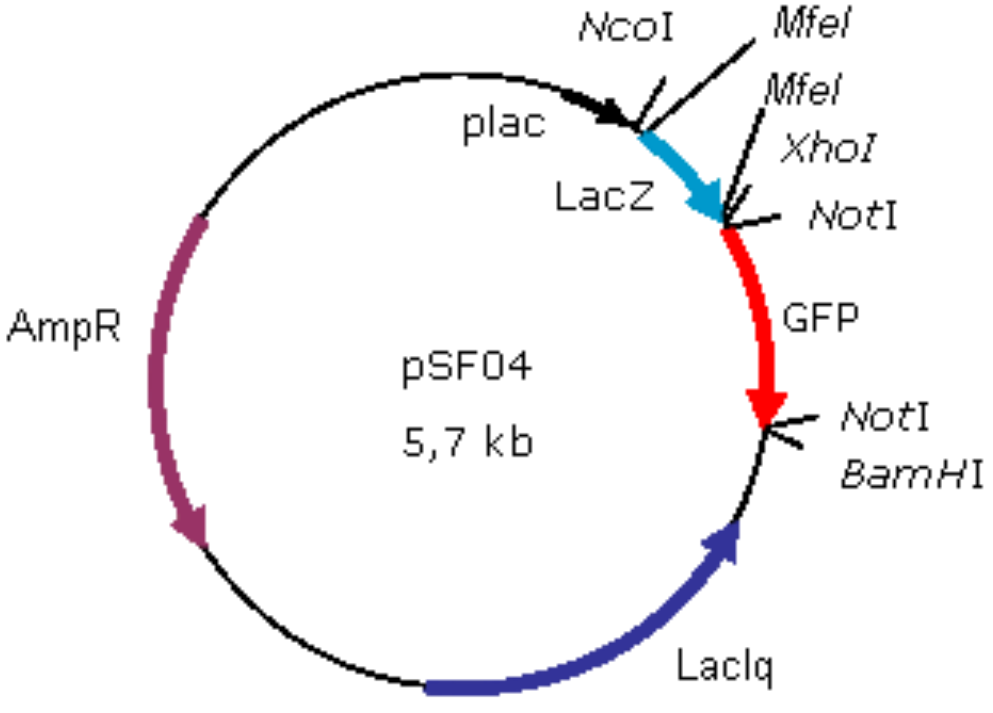

Supplement: Additional file 1 — pSF-04 expression vector map. [file 1472-6807-8-33-S1.png]

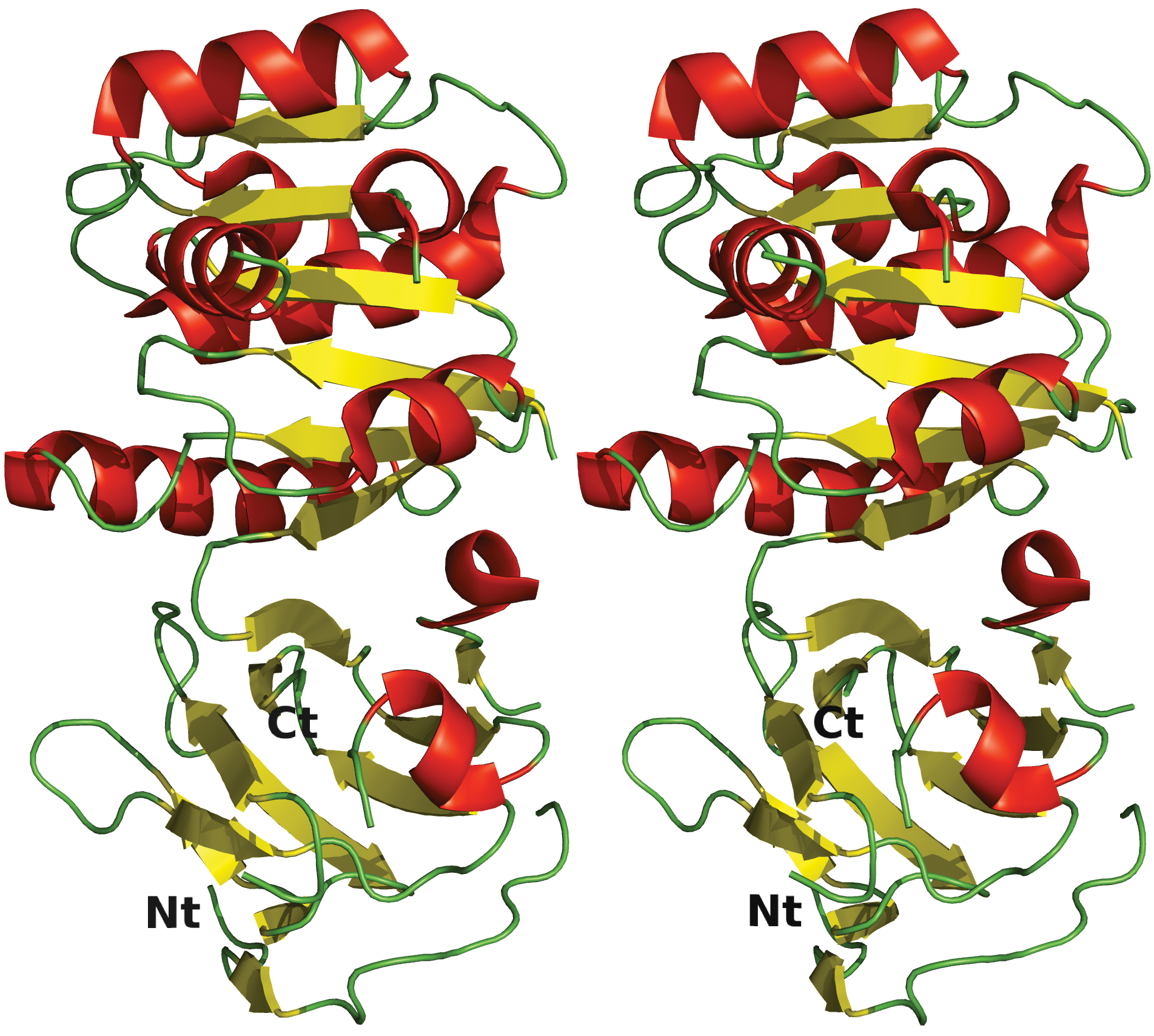

Supplement: Additional file 2 — Stereo ribbon diagram of the C. albicans TPK monomer structure color coded according to secondary structure elements (helices in red, β-sheet in yellow and loops in green). [file 1472-6807-8-33-S2.png]

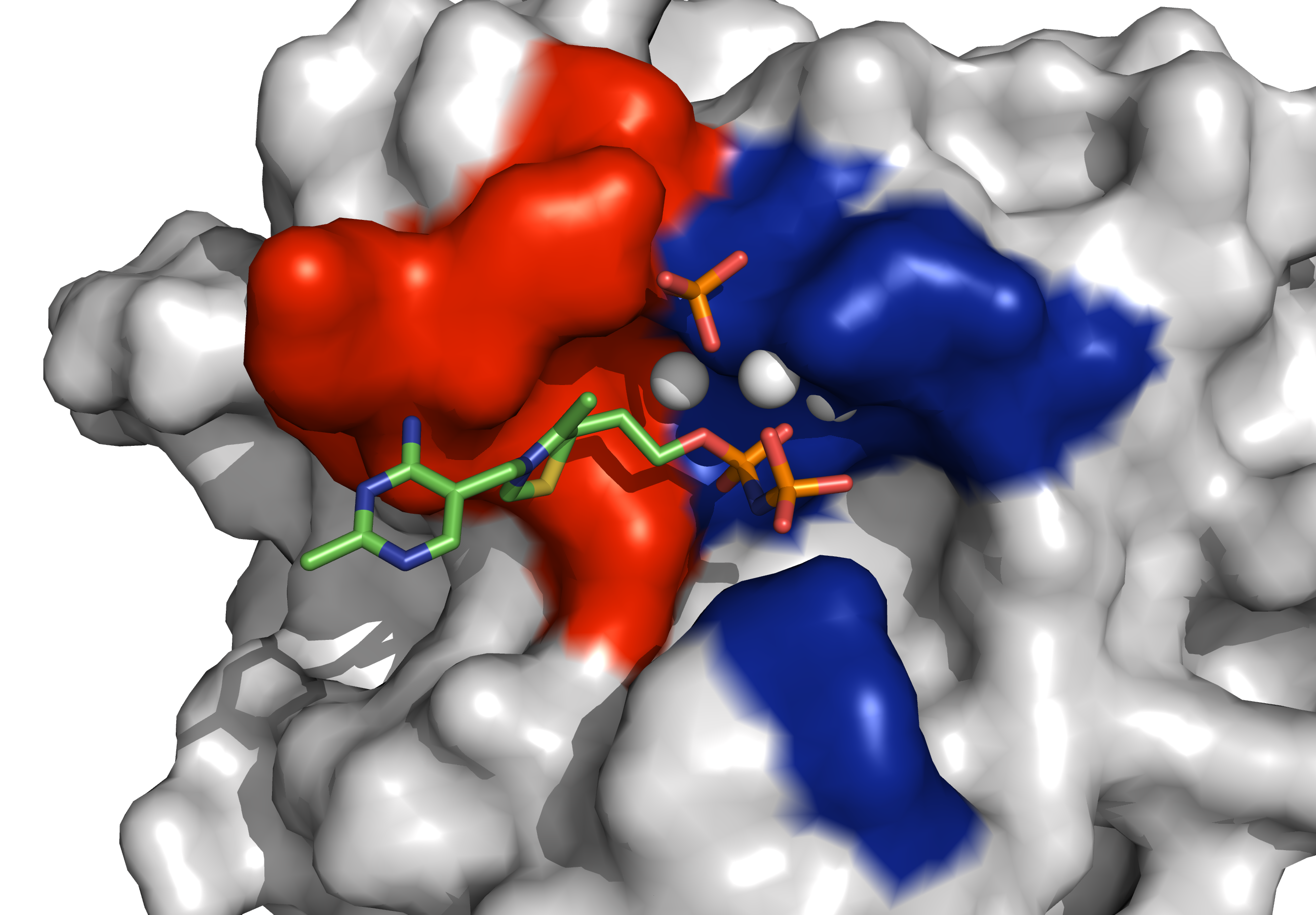

Supplement: Additional file 3 — Molecular surface representation of the C. albicans TPK active site. Conserved residues in contact with at least one ligand are colored in blue and variable ones in red. All other residues are colored in grey. The ligands are colored with the Pymol default colors and Mg2+ ions are in white. [file 1472-6807-8-33-S3.png]

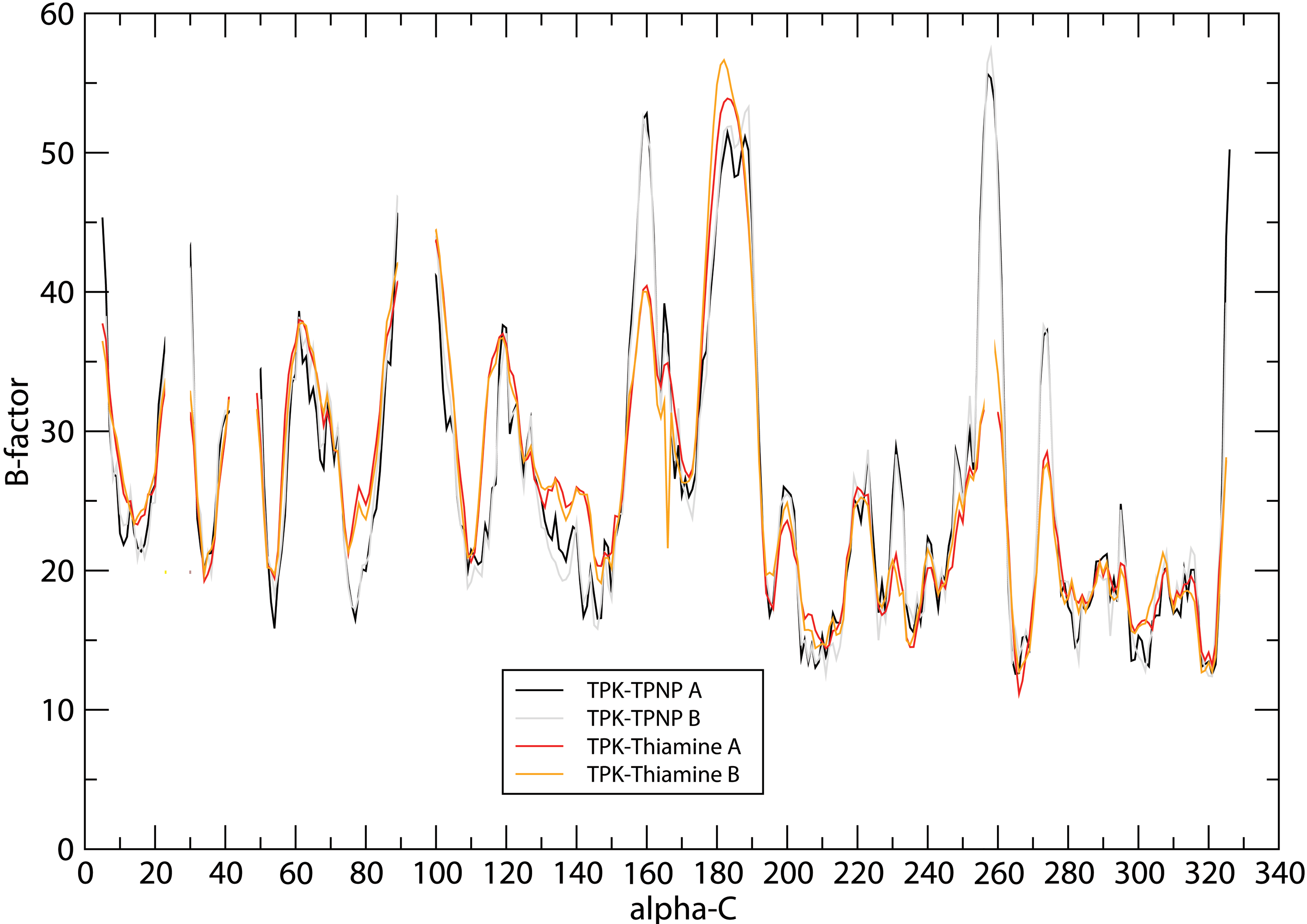

Supplement: Additional file 4 — Graph representation of the B-factors values of the C. albicans TPK structures in complex with thiamine and thiamine-PNP. Monomers A and B of TPK/Thiamine-PNP are shown in black and grey respectively. Monomers A and B of TPK/Tiamine are shown in red and orange respectively. [file 1472-6807-8-33-S4.png]
